# A pilot study evaluating use of a computer-assisted neurorehabilitation platform for upper-extremity stroke assessment

**DOI:** 10.1186/1743-0003-6-15

**Published:** 2009-05-28

**Authors:** Xin Feng, Jack M Winters

**Affiliations:** 1 Marquette University, Dept of Biomedical Engineering, Olin Engineering Center, Milwaukee, Wisconsin 53233, USA; 2 Lexmark International, 740 West New Circle Road, Lexington, Kentucky 40550, USA

## Abstract

**Background:**

There is a need to develop cost-effective, sensitive stroke assessment instruments. One approach is examining kinematic measures derived from goal-directed tasks, which can potentially be sensitive to the subtle changes in the stroke rehabilitation process. This paper presents the findings from a pilot study that uses a computer-assisted neurorehabilitation platform, interfaced with a conventional force-reflecting joystick, to examine the assessment capability of the system by various types of goal-directed tasks.

**Methods:**

Both stroke subjects with hemiparesis and able-bodied subjects used the force-reflecting joystick to complete a suite of goal-directed tasks under various task settings. Kinematic metrics, developed for specific types of goal-directed tasks, were used to assess various aspects of upper-extremity motor performance across subjects.

**Results:**

A number of metrics based on kinematic performance were able to differentiate subjects with different impairment levels, with metrics associated with accuracy, steadiness and speed consistency showing the best capability. Significant differences were also shown on these metrics between various force field settings.

**Conclusion:**

The results support the potential of using UniTherapy software with a conventional joystick system as an upper-extremity assessment instrument. We demonstrated the ability of using various types of goal-directed tasks to distinguish between subjects with different impairment levels. In addition, we were able to show that different force fields have a significant effect on the performance across subjects with different impairment levels in the trajectory tracking task. These results provide motivation for studies with a larger sample size that can more completely span the impairment space, and can use insights presented here to refine considerations of various task settings so as to generalize and extend our conclusions.

## Background

In the United States, stroke is the leading cause of disability and affects about 5.6 million individuals today, resulting in an estimated direct and indirect cost of $62.7 billion [1]. Up to 85% of the stroke survivors show initial upper extremity sensorimotor dysfunctions. Between 55% and 75% of patients continue to experience upper extremity functional limitations after 6 months of the stroke, which are associated with diminished health-related quality of life [2].

Quantification of upper-extremity movement features in patients with stroke is a critical component for supporting the optimization of intervention plans [3], so as for understanding the underlying mechanism of the upper extremity impairments induced by stroke. In today's rehabilitation practice, stroke assessment in clinical settings generally involves use of observer-based, ordinal scale instruments, such as the Functional Independence Measure (FIM) [4], Fugl-Meyer Assessment [5], Wolf Motor Function Test [6], Chedoke-McMaster Stroke Assessment [7] and so on. Although these ordinal instruments are well established and have proven to be reliable and sensitive for measuring gross changes in functional performance, they can be problematic because of poor consistency in the differences between scale increments [8]. They also lack sensitivity to characterize small yet potentially important changes during the intervention process [9,10]. The subjectivity of these tests is well recognized [11]. Furthermore, due to the economic pressure on the healthcare system, patients with stroke, particularly the outpatient population, have a limited access to rehabilitation resources [12]. Due to these reasons, there is a need to develop cost-effective, semi-autonomous/autonomous, yet sensitive assessment instruments for patients with stroke at home, which is characterized by low cost and under-supervision from rehabilitation practitioners.

Measures derived from kinematic trajectories associated with goal-directed tasks are continuous metrics which can potentially be sensitive to the subtle changes in the intervention process. They can also be more objective and repeatable across subjects than clinical ordinal scales [10]. The results from previous studies which examined the assessment capability of kinematic measures for stroke-induced impairments are summarized below.

Reaching to a target object is one fundamental component in the activities of daily livings (ADLs) (e.g. eating, drinking, grooming) which involve arm movements. Many studies have examined point-to-point reaching in subjects with stroke-induced impairments and found that their movements are characterized by slowness [13,14], spatial and temporal segmentation [15], abnormal patterns of muscle activation [13,16], decreased movement range [17] and so on. Selected kinematic measures developed by these studies, such as movement time, elbow extension, shoulder flexion, displacement of the trunk and the active ranges of motion (ROM), have shown a significant correlation with the clinical motor function scales (e.g. upper extremity motor control portion of Fugl-Meyer assessment, Chedoke-McMaster Stroke Assessment). Some of these kinematic measures, such as movement time, significantly correlated with clinical spasticity scales (e.g. Ashworth scale [18], modified Ashworth scale [19]).

Trajectory-tracking tasks require common components involved in both perception-action coupling and functional motor tasks: perception of environmental constraints, motor planning and execution, and corrective monitoring of performance including explicit feedback [10]). Several studies have already evaluated the assessment capability of trajectory tracking task with subjects with stroke-induced impairments. It has been demonstrated that the motor functional level of subjects and their performance in trajectory-tracking tasks are closely related [20,21]. Furthermore, certain kinematic metrics (e.g. root mean squared error (RMSE)) derived from trajectory tracking tasks have been demonstrated as a reliable, sensitive assessment tool of the upper-extremity motor function in subjects with stroke-induced hemiparesis [10].

Many daily activities, such as holding a cup of tea, driving a car, and replacing light bulbs, require one to cope with some level of instability in the manipulated object. It is important to evaluate the performance of subjects with stroke in a goal-directed task in an unpredictable mechanical environment to better understand the strategy that they used to cope with instability [22,23]. Recent experimental evidence also suggests that patients with stroke-induced impairments may likely benefit from training of the paretic limb in unpredictable mechanical environments [24-27], and the improvement can potentially be transferred to ADLs.

These studies laid down a rationale stage for developing kinematic measures derived from goal-directed tasks as upper-extremity assessment instruments, but also leave several fundamental questions unanswered. First, to date the majority of biomechanical upper-extremity evaluations involve reaching and trajectory tracking performed at a limited number of task settings, most commonly at one speed in the horizontal plane with the arm supported [28]. In order to personalize the intervention plan for a given client with stroke-induced impairments with the goal of optimizing the functional outcome, we need to better understand the performance of subjects with stroke under various goal-directed tasks. It is necessary to develop a suite of performance metrics to characterize various movement features in the goal-directed tasks, such as slowness, segmentation, and a decreased range of motion, movement speed, and coordination and so on.

Second, while interventions that are based on robotic therapy have proven to be effective for sub-acute and chronic stroke populations [29-31], the advantages of mechanical guidance by the robotic device over other stroke therapy technique still remain elusive [32]. It is worthwhile to examine the performance of subjects with stroke under various mechanical environments in goal-directed tasks, so that we can better understand the role of force on the performance of subjects with stroke-induced impairments and potentially optimize the mechanical environment in the robotic-assisted therapy plan for a given client.

Third but probably more important for outpatient rehabilitation, most of these studies summarized here are using either large robotic systems or three dimensional (3D) marker-based motion analysis systems as their research platform. While these tools provide abundant sensor-based performance data, high costs and mechanical sophistication appear to limit the likelihood of their large-scale implementation, particularly for the home setting, which is more convenient and sometimes the only option for many persons who could benefit from therapeutic interventions.

In summary, there is a need to develop alternative, cost-effective yet still sensitive tools for upper-extremity stroke assessment, particularly for outpatient rehabilitation. This paper presents the findings from a pilot study using UniTherapy software [33,34] interfaced with a conventional force-reflecting joystick. This software also has been used by adapted larger joysticks called TheraJoy [35-37] and for driving wheels called TheraDrive [38], but with different aims. Here the focus is on evaluating a suite of performance metrics that were derived from goal-directed tasks supported by UniTherapy technology. The sensitivity of these metrics as home-based assessment instruments were evaluated within the context of two hypotheses: hypothesis 1) Impairment level of human subjects influences performance on various goal-directed tasks using a conventional force-reflecting joystick, and hypothesis 2) Force field settings in continuous tracking tasks influence the performance of human subjects across impairment levels. The focus here is whether our performance metrics, developed using a low-cost computer-assisted platform, have as enough usability and sensitivity for use as assessment tools for a home rehabilitation as a component within a larger-scale biomechatronic system. A key question relates to which of the many viable metrics are most effective in terms of sensitivity, here addressed within the context of these hypotheses, and this is the focus of the discussion.

## Methods

In this section, we describe the experiment setup and protocols used in this study, particularly focusing on the selected goal-directed tasks for evaluating the potential of UniTherapy software interfaced with the conventional force-reflecting joystick for upper-extremity stroke assessment.

### Experiment platform

We utilize UniTherapy software interfaced with a conventional force-reflecting joystick (Microsoft Sidewinder) along with TheraJoy (adapted joystick) [35-37] for the data collection component of in this study. UniTherapy software implements three toolboxes consisting of customizable goal-directed tasks to quantify the various aspects of upper-extremity movement features [33]. These toolboxes are outlined below:

• The Range of Capacity (ROC) toolbox can be used to assess the user's initial and final capability ROM when using an input device and optionally used to map between the input device workspace range and the user's capability range by a two dimensional (2D) transformation algorithm [33].

• The Tracking toolbox implements discrete tracking and continuous tracking. Discrete tracking (target acquisition) requires the subjects to move a cursor into a target window with accuracy; once the subjects get into the target window, they receive a positive visual feedback and optionally a sound cue, and they are required to stay as stable as possible for a threshold of success time (defined as dwelling time); after successful completion of dwelling time, the target jumps to the next predefined position. Continuous tracking instructs subjects to follow the continuously moving target and try to stay within the target window as much as they can, for which they receive a positive visual feedback when they stayed within the target window. The size of the target window and dwelling time are customizable.

• The users' stable motor performance is also evaluated using the System Identification toolbox. Predefined force perturbations are applied to the subject under a certain instruction (e.g. "hold," "relax"). The force data and experimenter's instruction are recorded as input while subject's movement data is recorded as output.

UniTherapy applied none or varying levels of force-feedback to physical therapeutic interfaces, depending on the settings and the task; these were derived from a series of force effects such as spring, damper, constant and so on in DirectX. Both sampling of position data and the input of force were at 33 Hz.

The joystick systems used in this study consisted of the conventional force-reflecting joysticks (Microsoft Sidewinder) and the larger "TheraJoy" in horizontal and vertical settings [35-37], and incorporate a larger range of motion that can be scaled and modified depending on the anthropometrics and abilities of the user. Here the focus is on a detailed analysis of selected data for the conventional joystick, related to the aim of identifying sensitive assessment metrics; most of the other data was used as part of the Master's Thesis by Johnson [37].

There are multiple reasons for this focus on the conventional force-reflecting joystick. First, unlike the larger custom-made TheraJoys, these joysticks are available without special alteration. Second, it has been shown through video analysis that significant movement of the torso was uncommon when using the conventional joystick versus the TheraJoy devices [37]. Third, EMG analysis has shown convincingly that the shoulder muscles are quite involved with the conventional joystick because the high degree of humeral rotation that accompanies "horizontal" movements and the natural reach involved in "vertical" movements – movement-related EMG activity for the pectoralis major, anterior deltoid and lattissimus dorsi was consistently higher than for the wrist flexor and extensor groups, and indeed even the triceps and posterior deltoid were typically more active than the wrist flexors [37]. Fourth, the forces applied by the joystick motor to the hand are much higher for the conventional joystick, with its smaller lever arm. Fifth, the system bandwidth due to applied force oscillations is higher with the lower mass of the Microsoft Sidewinder joystick, about 9 Hz, with there still being plenty of movement response up to the upper limit of 16 Hz, and with reliable linearity of the output torque [38]. Finally, a systematic study of task performance with the conventional joystick placed at 6 different locations within the workspace showed only moderate performance variance within the primary ability space of the user [37], supporting the decision made for this study of letting the subject select a location that they found to be a comfortable range, given their abilities.

### Performance metrics

A number of customized and standard performance metrics examining accuracy [20,21,13,10,39], smoothness [15,17], response capability [14,40], movement quickness [13-15], curvature [13,27,40], steadiness, strength[41], exercise intensity and duration, motivation [42], and so on have been developed for each toolbox in UniTherapy [34]. These metrics were implemented to quantify performance outcomes of goal-directed tasks, monitor training intensity and evaluate patients' adherence to the protocol [34,38]. Table 1 summarizes selected performance metrics that are used in the analysis of the goal-directed tasks presented in this paper.

**Table 1:** Summary of the performance metrics for the goal-directed tasks

Goal-directed Tasks	Metric	Definition	Remark
Continuous Tracking	Percentage Time in Target (PTT)	The percentage of the time the human subject staying within the target window.	Accuracy and Steadiness
	
	Root Mean Square Error (RMSE)	The squared root of the mean-squared distance from the subject position to the target position.	Accuracy
	
	Deviation	The mean of the perpendicular distance from the subject position to the target line within the movement time.	Path Deviation
	
	Speed_Mean (SM)	The mean of the subject speed during the continuous tracking task	Speed Consistency
	
	Speed_StdDev (SS)	The standard deviation of the subject speed during the continuous tracking task	Speed Consistency

Discrete Tracking (Target Acquisition)	Reaction Time	The time from the movement of the target to the first significant movement made by the subject.	Reaction Quickness
	
	Movement Time	The time window from the end of the reaction time to the time after which the user has stably reached the target.	Movement Quickness
	
	Deviation	The mean of the perpendicular distance from the subject position to the target path line.	Path Deviation
	
	Movement Speed	The mean of subject speed.	Movement Quickness
	
	Peak Speed Number	The number of local maximum speed within the movement time window.	Smoothness
	
	Dwelling Percentage Time in Target	The percentage of the time that the subject stayed within the target window during the *Dwelling Time*.	Stability
	
	Success Percentage	The percentage of the targets that have been successfully reached by the subject.	Overall Reaching Capability

System Identification	Error_Mean	The mean of the displacement outside of the holding area in both x and y direction	Strength (under "hold" instruction)
		
	Error_StdDev.	The standard deviation of the displacement outside of the holding area in both x and y direction	

### Subjects

This study was approved by the Institutional Review Board (IRB) at Marquette University. Subjects with stroke-induced hemiplegia (n = 9) and able-bodied (Control) subjects (n = 8) participated in this study and gave informed consent. The controls were not age-matched, and consisted of a convenience sample of mostly young adults. Given that our overriding aim related to assessment metric sensitivity rather than hypothesis testing, the primary objective for including controls was to establish a normal baseline for the various performance metrics, Table 2 summarizes the information of the subjects with stroke-induced disability, all of who were at least twelve months post-stroke and had been discharged from all forms of physical rehabilitation. The upper extremity motor control portion of the Fugl-Meyer (UE-FM) assessment test was included as a tool to assess level of upper-extremity motor impairment of subjects with stroke. UE-FM is used to further partition subjects with stroke into a low (n = 4, UE-FM (22 to 57)) and high (n = 5, UE-FM (63 to 66)) functional subgroup. Of note is that a UE-FM of 66 is the same score as for an able-bodied normal person, and yet these individuals clearly viewed themselves as disabled; the lack of sensitivity of metrics such as UE-FM – a ceiling effect in this case – was a key motivation behind this study.

**Table 2:** Summary information of the subjects with stroke

Subjects Group	Subject ID	Age	Gender	Impaired side	UE-FM	Sub-group
Stroke-Induced Hemiplegia	S1	55	F	R	22	Low function
		
	S2	33	M	R	24	
		
	S3	76	F	L	46	
		
	S4	57	F	L	57	
	
	S5	58	F	R	63	High function
		
	S6	58	F	L	65	
		
	S7	58	M	R	65	
		
	S8	55	F	L	66	
		
	S9	58	M	L	66	

Note: F: Female, L: Left, M: Male, R: Right, UE FM: upper-extremity motor control portion of Fugl-Meyer assessment.

### Procedures

The experimental protocol consisted of two sessions focusing first on training the individual on using each device (conventional joystick (CJS) and TheraJoy in horizontal (HJS) and vertical (VJS) settings), then on collecting performance data for a suite of goal-directed assessment tasks in the second session. In the first session, all joysticks were placed in the position of greatest comfort for the subject, including altered handle position and interface to allow for maximum comfort. Subjects first completed several goal-directed tasks from the Tracking and System Identification toolbox with the conventional joystick. A subset of tasks was then repeated with the HJS and VJS. The medial-lateral and proximal-distal direction of the joystick movements were mapped to the X and Y direction on the computer screen. All tasks were repeated with both arms. On the second day, these goal-directed tasks were repeated but this time both video and EMG data were also collected but not presented here.

### Tasks

Representative results from three classes of task are presented here: 1) continuous circle tracking tasks under three different force settings (e.g. white noise perturbation, no force, spring-assistance), 2) eight-point rectangle target acquisition, and 3) pseudo-random perturbation task under "hold" instruction. Both the control and stroke groups were asked to complete these tasks. For both continuous tracking and target acquisition tasks, the target window size were set at 5% of the width of the workspace; for the target acquisition task, the dwelling time for successful completion was set to one second.

#### Continuous Circle Tracking

Here subjects were asked to follow a continuously moving target along a circle pattern and to stay within the target window as much as possible. The circle pattern was screen-centered with a diameter equalling 90% height of the workspace. The target smoothly moved with a speed 12 seconds/circle in the counter-clockwise direction. They completed this tracking task under three conditions: spring-assistance (eq. 1), white noise perturbation with bandwidth frequency content up to 16 Hz (eq. 2) and no force. The force field of spring assistance and white noise perturbation are generated by:

(1)

(2)

where F_*x*, *y *_represents the force at *x *and *y *directions, K represents the spring coefficient and was set as the highest stiffness which the conventional joystick can provide, Random [0,1]_*x*, *y *_is a random function on both x and y direction separately (with values of 0 as no force and ± 1 as maximum force magnitude), Subject_x, y _represents the subject position, and Target_x, y _represents the target position. The spring-assistance force vector was directed to help pull the subject toward to current target location.

**Figure 1:**
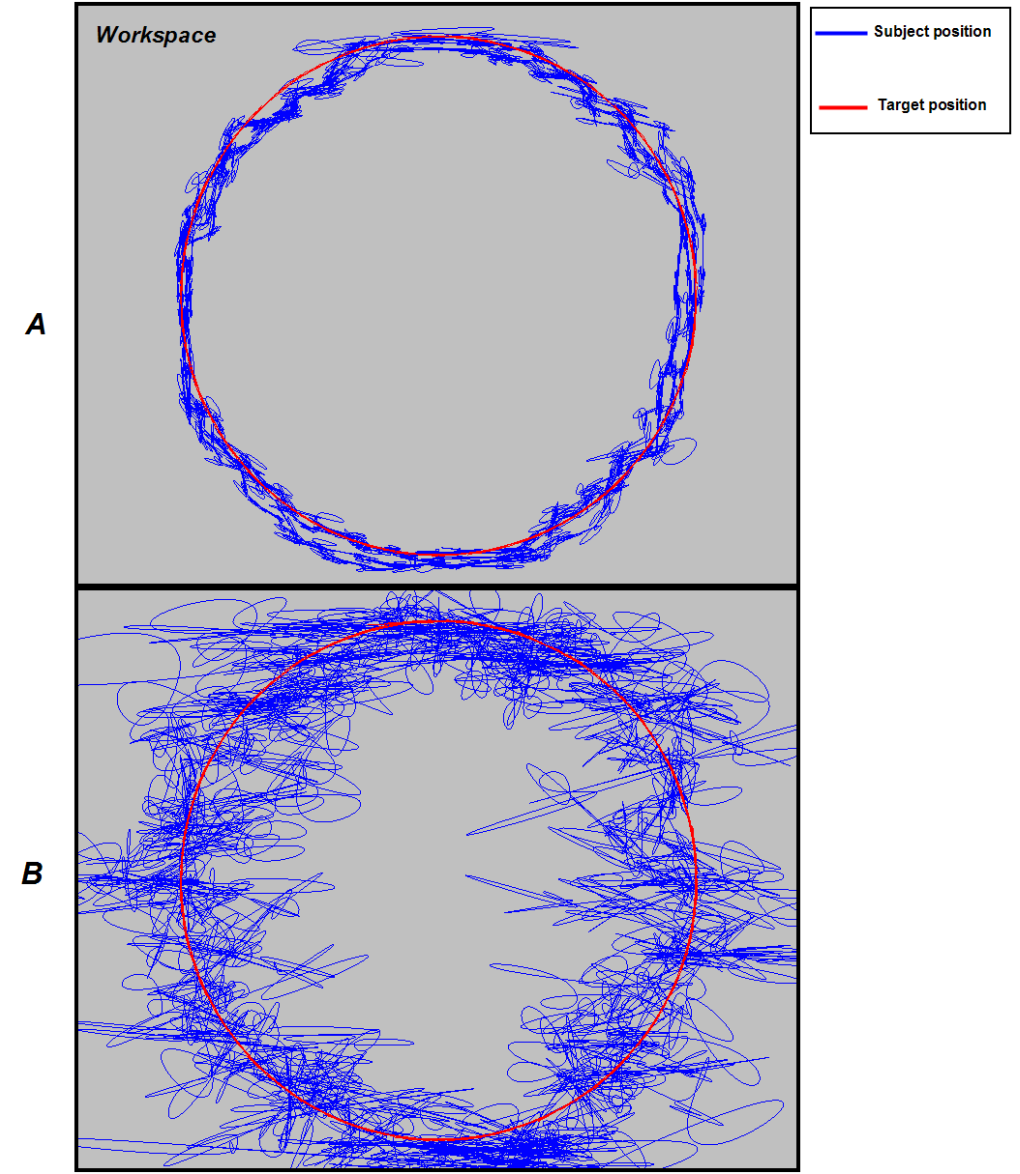
**Example results of the continuous circle tracking task using a conventional force-reflecting joystick**. The data is from able-bodied subject 1012. Note: *A*: spring assistance, *B*: white noise perturbation force field, *blue color*: subject position; *red color*: target position.

The task was repeated 3 times under each type of force field, with the sequence of force field being randomly arranged by the experimental protocol in order to minimize the "learning effect" on the result. Example results are provided in Fig. 1.

#### Eight-Point-Rectangle Target Acquisition

**Figure 2:**
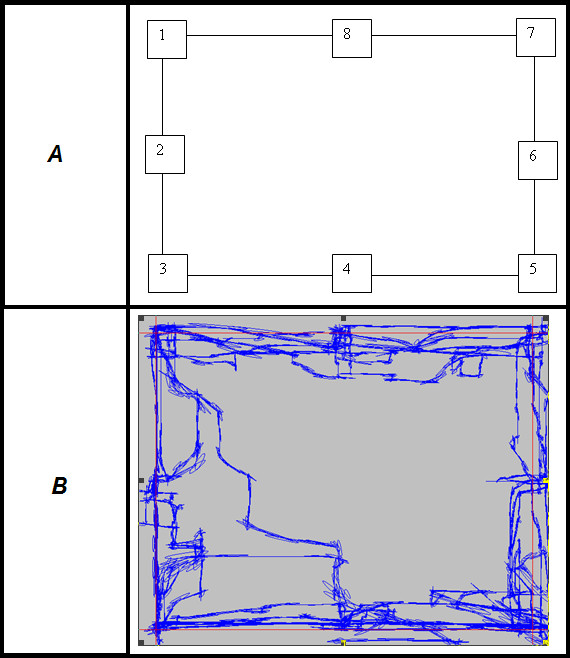
**Eight-point-rectangle Tracking Pattern used for a Target Acquisition Task**. *A*: Eight-Point-Rectangle track pattern used in the target acquisition task. The rectangle is screen-centered with 90% width and height of the workspace. The numbered rectangle represents the sequence of predefined target position, which is equally distributed on four lines of rectangle. *B*: Example subject position and target position data for Eight-Point-Rectangle target acquisition task (from subject 1006 in low functional stroke group). Note: *blue color*: subject position; *red color*: target path line.

Subjects were asked to complete an Eight-Point-Rectangle target acquisition task, where they moved the conventional joystick to acquire a square box (target window) with accuracy and at a comfortable speed. As shown in Fig. 2, the rectangle pattern was screen-centered with 90% width and height of the workspace. The target moved to eight locations which appeared in a counter-clockwise order and were equally distributed on the four lines of rectangle. This rectangle pattern was repeated three times. The task is space-predictable, as the subject knew the location for the next target; the time between target transitions was not purely predictable, as it depended on the subject's performance in reaching and staying in the target window.

#### Pseudo-Random Perturbation under "hold" instruction

**Figure 3:**
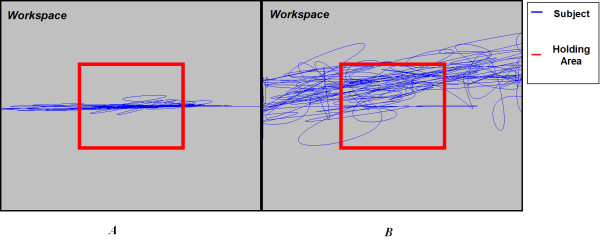
**Example Data from the Pseudo-random Perturbation at X and Y Directions Separately under "Hold" Instruction**. The position data are from *A*: subject 1011 (able-bodied subject) and *B*: subject 1005 (subject with low functional stroke). Note: blue: subject position; red: holding area.

As shown in Fig. 3, subjects were asked to complete a pseudo-random perturbation task generated by the System Identification toolbox, with the conventional force-reflecting joystick under "hold" instruction, in which they were asked to stay within the holding area during the perturbation as much as possible. The pseudo-random perturbation was generated by an algorithm which ensured that the amplitude of the force has an equal opportunity to be set among the negative maximum value, 0, and the positive maximum value, generating frequency content up to 16 Hz. The task involved application of these pseudo random perturbations in each of the x and y directions for three seconds separately. The start time and sequence of the perturbation appeared unpredictable to the subjects, and of a magnitude that made it challenging for the "hold" instruction. The holding area in this task was thus large, consisting of a screen-centered rectangle with 40% width and height of the workspace.

### Data and statistical analysis

Representative tasks were analyzed across subjects using the performance metrics defined in Table 1. Mean and standard deviation values were calculated and presented for control (n = 8), high function (n = 5), and low function (n = 4) groups. For the continuous circle tracking task, a mixed-design repeated measure ANOVA test was used to test between group (by functional level) and within group (by force settings) difference. For the eight-point rectangle target acquisition and pseudo-random perturbation tasks, a repeated measure ANOVA test was used to test between group (by functional level) differences. The Tukey test was used for post-hoc analysis. A significance threshold level of p < 0.05 was used for interpretation. Statistical analysis was performed on the data using XLSTAT 2006 (AddinSoft, ).

## Results

### Continuous circle tracking under various force field

Table 3 provides the means and of performance metrics for continuous circle tracking tasks (e.g. Percentage Time in Target (PTT), Root Mean Square Error (RMSE), Deviation, Speed_Mean (SM), Speed_StdDev (SS)) under conditions of white noise perturbation, no force and spring-assistance force fields across all subjects. For between group difference, the results for all of these metrics show significant differences between low functional stroke group and controls/high functional stroke group, which suggests that the performance of able-bodied/high functional stroke subjects in the trajectory tracking tasks tend to be more accurate (PTT, RMSE), stable (PTT), with less path deviation (Deviation) and better speed consistency (SM, SS) than subjects with low functional stroke. There is also a significant difference with SS metric and a strong trend in the differences with PTT (p = 0.149) and SM (p = 0.105) metrics between control and high functional stroke group, which suggest that the performance of able-bodied subjects in the trajectory tracking task tend to be more accurate (PTT), stable (PTT) and with better speed consistency (SM, SS) than high functional stroke subjects.

**Table 3:** The mean and standard deviation of the performance metrics in the continuous circle tracking tasks

	By Functional level			By force settings		
	Control	High	Low	Assistance	No force	Perturbation

PTT	40.87 ± 23.00†	31.80 ± 17.70†	14.30 ± 16.19‡	50.10 ± 23.94‡	33.28 ± 12.39‡	13.04 ± 6.63‡

RMSE	5.02 ± 2.47†	6.25 ± 2.16†	19.29 ± 20.96‡	6.58 ± 8.71	7.54 ± 2.44	12.99 ± 16.05

Deviation	1.13 ± 1.14†	1.11 ± 0.83†	9.57 ± 15.18‡	1.94 ± 5.56	2.68 ± 0.78	5.64 ± 11.50

SM	1.93 ± 1.19†	2.39 ± 1.69†	3.23 ± 2.37‡	1.17 ± 0.08†	1.31 ± 0.24†	4.53 ± 1.09‡

SS	1.37 ± 0.87‡	1.94 ± 0.99‡	2.85 ± 1.68‡	0.89 ± 0.35‡	1.54 ± 0.75‡	3.14 ± 1.10‡

The continuous circle tracking tasks are repeated under white noise perturbation, no force and spring assistance force field. The results are grouped by functional levels and force settings. Note: PTT: percentage time in target, RMSE: root mean square error, SM: Speed_Mean; SS: Speed_StdDev, †: Differences significant from another group (p < 0.05). ‡: Differences significant from another two groups (p < 0.05).

For within group difference, there are significant differences with PTT and SS metrics between spring-assistance, no force and white noise perturbation settings. This suggests that spring-assistance can significantly improve the performance on accuracy, steadiness and speed consistency in the trajectory tracking across subjects with different impairment levels, while perturbation significantly worsens these aspects of movement performance. There is also a significant difference with SM metric between perturbation and no force/assistance setting, which confirms that perturbation significantly diminishes the capability of keeping consistent with the target speed in the trajectory tracking tasks across subjects.

### Eight-point rectangle target acquisition

**Figure 4:**
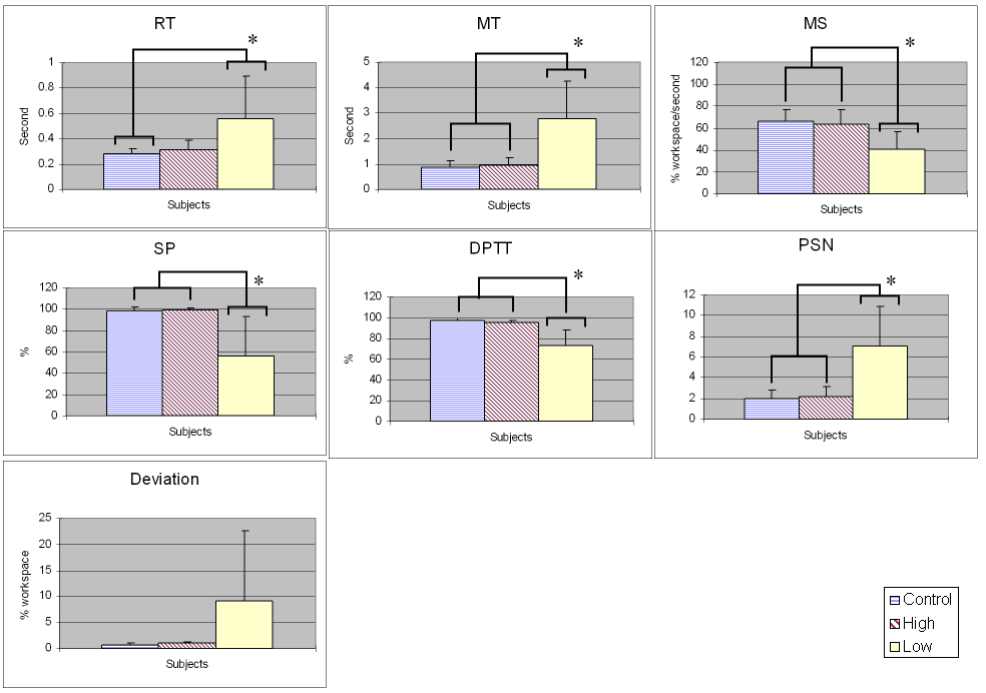
**The means and standard deviation of the performance metrics in eight-point rectangle target acquisition task across subjects**. The results are grouped into control, high functional stroke and low functional stroke groups. Asterisks indicate significant differences between groups at P < 0.05 (Tukey test). Notes: DPTT: dwelling percentage time in target, MS: movement speed, MT: movement time, PSN: peak speed number, RT: reaction time, SP: success percentage.

Fig. 4 provides the means and standard deviation of strategic discrete-task performance metrics [Reaction Time (RT), Movement Time (MT), Deviation, Movement Speed (MS), Peak Speed Number (PSN), Dwelling Percentage Time in Target (DPTT) and Success Percentage (SP)] for the eight-point rectangle target acquisition task across the subjects. These metrics are defined in Table 1. The results on RT, MT, MS, PSN, DPTT and SP metrics show that significant differences exist between low functional stroke group and controls/high functional stroke group, which suggest that the performance of able-bodied/high functional stroke subjects have higher capabilities in the aspects of reaction quickness (RT), movement quickness (MT, MS), smoothness (PSN), steadiness (DPTT) and overall reaching (SP). There is no significant between-group difference shown in Deviation metric, which suggest that this metric is not sensitive enough in target acquisition tasks to differentiate the subjects with different impairment levels.

### Pseudo-random perturbation under "hold" instruction

**Figure 5:**
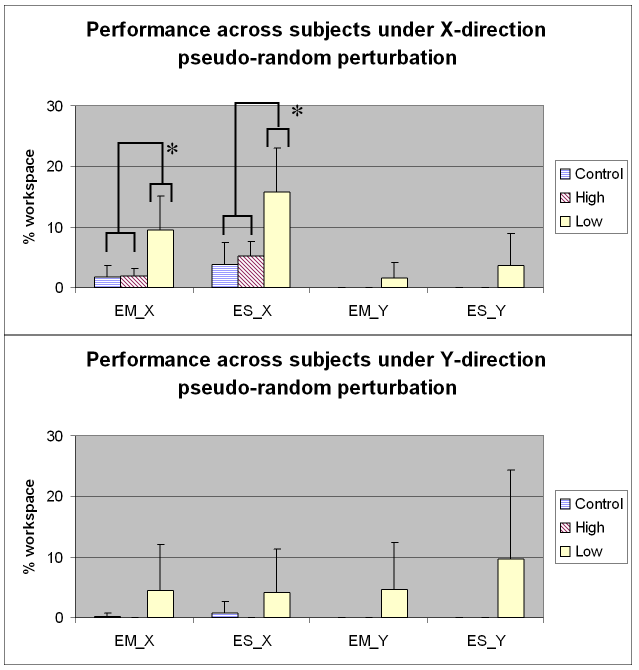
**The means and standard deviation of the performance metrics in x and y direction pseudo-random perturbation separately, under "hold" instruction**. The results are grouped into control, high functional stroke and low functional stroke groups and normalized to % workspace width. Asterisks indicate significant differences between groups at P < 0.05 (Turkey test). Notes: EM_X: Error_Mean at x direction, EM_Y: Error_Mean at y direction, ES_X: Error_StdDev at y direction, ES_Y: Error_StdDev at y direction.

Fig. 5 provides the means and standard deviation of performance metrics for the pseudo-random perturbation tasks [Error_Mean at x direction (EM_X), Error_StdDev at x direction (ES_X), Error_Mean at y direction (EM_Y), Error_StdDev at y direction (ES_Y)], in *x *and *y *directions separately, under "hold" instruction. These metrics are defined in Table 1. Under the *x*-direction pseudo-random perturbation, EM_X and ES_X metrics showed a significant difference between low functional stroke group and controls/high functional stroke group, which suggests that it is challenging for patients with low functional stroke to compensate the perturbation from medial-lateral direction. In general there was more motion and presumably less impedance in this direction. Under the *y*-direction pseudo-random perturbation, there is no significant between-group difference shown in EM_Y and ES_Y metrics. Under *x *and *y *direction pseudo-random perturbation separately, there is no *y *direction error for control and high functional stroke group. These data suggest that subjects with different impairment levels, including low functional stroke, are able to compensate for pseudo-random perturbations in the proximal-distal (or toward-away) direction much better than in the medial-lateral (or left-right) direction.

## Discussion

This paper evaluates the assessment capabilities of the UniTherapy software interfaced with the conventional force-reflecting joystick, using selected goal-directed tasks which were designed to capture the basic movement components (e.g. reaching, tracking, and coping with instability) that may relate to ADLs. A suite of kinematic measures were developed to examine various movement features in each type of goal-directed tasks. The results support the potential of using UniTherapy software with joystick system as an upper-extremity assessment instrument. We demonstrated the ability of using various types of goal-directed tasks to distinguish between subjects on different impairment levels (hypothesis 1). In addition, we were able to show that different force fields have a significant effect on the performance across subjects with different impairment levels in the trajectory (continuous) tracking task (hypothesis 2).

For assessment measures associated with the continuous tracking tasks, for the continuous circle tracking task, we found that certain measures can differentiate between control/high functional group and low functional stroke group: performance of able-bodied/high functional stroke subjects in the trajectory tracking tasks were significantly more accurate (Percentage Time in Target (PTT), Root Mean Square Error (RMSE)), stable (PTT), with less path deviation (deviation) and better speed consistency (Speed_Mean (SM), Speed_StdDev (SS)) than subjects with low functional stroke. This was true even for the relatively small size of the sample population, as reflected in the levels of statistical significance between groups. One possible reason for this performance difference was that subjects with moderate to severe stroke may take more time for movement planning and for correction based on their visual feedback. In comparing between control and high functional stroke group, there is also a significant difference on SS metric as well as a trend of difference observed on PTT and SM metrics: able-bodied subjects can perform more accuracy (PTT), more steadily (PTT) and with better speed consistency (SM, SS) than subjects with high functional stroke. The capability to differentiate between able-bodied subjects and subjects with high functional stroke as well as between high and low functional stroke subjects by using SS metric and potentially the PTT and SM metrics suggests that these metrics could be sensitive to the impairment level of human subjects across assistance, no force and perturbation force settings with the trajectory tracking task. The result on the RMSE metric was consistent with other published data that RMSE were found to be sensitive the impairment level of human subjects [20,21,10]. However, as is reflected in the paragraphs that follow, overall this most convenient metric was not found to be one of the more robust metrics in terms of sensitivity.

For assessment measures associated with discrete tracking tasks, for the eight-point rectangle task, the results for Reaction Time (RT), Movement Time (MT), Movement Speed (MS), Peak Speed Number (PSN), Dwelling Percentage Time in Target (DPTT) and Success Percentage (SP) metrics show that significant differences exist between control/high functional stroke group and low functional stroke group. This suggests that the able-bodied/high functional stroke subjects have higher movement capabilities in reaction quickness (RT), movement quickness (MT, MS), smoothness (PSN), stability (DPTT) and overall reaching (SP).

For assessment measures associated with perturbation with "hold" instruction tasks, under x-direction pseudo-random perturbation, the EM_X and ES_X metrics showed significant difference between low functional stroke group and controls/high functional stroke group, which suggest that it is challenging for patients with low functional stroke to compensate for the perturbation from the medial-lateral direction. This seems likely due to the directional differences in the spring-like impedance field within the horizontal arm workspace, with the stiffness value generally higher in the proximal-distal direction than the medial-lateral direction as documented by the previous studies [43-45]. Previous studies have also shown that shoulder stiffness is direction dependent and task dependent [46,47]. Under *y*-direction pseudo-random perturbation, there is no significant between-group difference shown in EM_Y and ES_Y metrics. This suggests that subjects with different impairment level, including low functional stroke, either can compensate for the pseudo-random perturbation from the proximal-distal direction or there are intrinsic direction-sensitive mechanical impedance reasons that are helpful, or most likely a combination of both. Of note is that the control and high functional stroke group can compensate quite well. In terms of functional assessment strategies, these results suggest that when using the perturbation task under the "hold" instruction as a strength assessment test, perturbations in the medial-lateral direction provide a more sensitive task setting (and associated metrics) than perturbations in the proximal-distal direction, and can potentially be a challenging task for the stroke subjects with weak strength.

In summary, despite the relatively small sample size, kinematic metrics developed for various types of goal-directed tasks in UniTherapy were able to differentiate subjects with different impairment levels, supporting hypothesis 1. This is consistent with previous findings by other research studies [20,21,13,17,10]. Particularly, SS, and potentially PTT and SM metrics, derived from the continuous tracking task, showed the best capability for differentiating between subjects with low function stroke and high function stroke, as well as between subjects with high functional stroke and able-bodied subjects.

When we tested three different force setting (e.g. white-noise perturbation, no force, spring-assistance) across subjects in a continuous circle tracking task, significant differences were shown by the PTT and SS metrics between spring-assistance, no force and white noise perturbation settings, thus supporting hypothesis 2. This suggests that spring-assistance can significantly improve the performance on accuracy (PTT), steadiness (PTT) and speed consistency (SS) in the trajectory tracking across subjects with different impairment level, while perturbation significantly worsens these aspects of movement performance. These results also confirm that perturbations significantly worsen the capability of keeping consistent (SM) with the target speed in the trajectory tracking tasks across subjects. Also, these results suggest that PTT emerges as a potentially sensitive assessment metric for trajectory tracking tasks across various task settings, since PTT has the capability to characterize different phases in the trajectory tracking task, including motor planning, motor execution, and movement correction based on the visual feedback [10]. While showing that perturbation force is challenging across subjects during the continuous tracking tasks, it has been suggested by other studies that persons with stroke-induced impairments may likely benefit from this type of "error augmentation" training of the paretic limb in unpredictable mechanical environments, and potentially that improvement can be transferred to ADLs [24,25].

The main limitations of this pilot study relate to the relatively small subject sample size. and also that the motor impairment level of our stroke population, as measured by the upper-extremity Fugl-Meyer score, was polarized in that we did not fully span the impairment workspace. Despite these limitations, our results suggest that the UniTherapy system and force-reflecting joystick tracking tasks in general have great potential for being used as a sensitive upper-extremity assessment tool. For instance, we saw clear evidence of a ceiling effect in the UE-FM in that we identified assessment metrics that could strongly delineate between stroke subjects with near-normal UE-FM scores and the normal population. Also of note is that a narrower in scope follow-up pilot study that more fully spanned the Fugl-Meyer score space and systematically considered the effects of task settings for force and speed magnitudes, but only for a subset of the class of continuous tracking tasks described here, confirmed that the force input settings and the tracking speed used in this study were within the range of effective choices [34].

The capability of examining basic upper-extremity movement assessment metrics periodically (and more frequently) can help optimize the intervention plan in order to yield functional benefits for a given client. While not emphasized here, the UniTherapy technology also supports telerehabilitation connections between a "telepractitioner" and "home client" which can extend the use of this platform into the scope of intervention [33,34]. Finally, a larger longitudinal study is still needed to evaluate these systems so as to reach a more general conclusion related to the sensitivity of these metrics to measuring subtle, yet important changes during an ongoing intervention process.

## Conclusion

This study, involving use of the UniTherapy software interfaced with a conventional force-reflecting joystick, validated the viability of using the combination of goal-directed tasks with associated kinematic metrics to obtain sensitive upper-extremity performance metrics. The results show that the UniTherapy platform can potentially be a sensitive upper-extremity assessment tool: it shows significant differences between low function and high function stroke subjects as well as high functional stroke and able-bodied subjects by using selected goal-directed tasks and kinematic metrics. This also helps inform other research groups on the most sensitive types of assessment metrics.

It is suggested that to get potentially more sensitive assessment results, the type of goal-directed task, the task settings and the kinematic metrics should be carefully selected, and based to some extent on a given client's impairment level and motor deficit. We also found that in the trajectory tracking task, mechanical assistance by our simple robotic device significantly improved the tracking performance of subjects across impairment levels, while perturbation significantly worsened it. Studies with a larger sample size with subjects in a spanned impairment space, and with considerations of various task settings, are necessary to generalize our conclusions and broaden the scope of application.

## Competing interests

The authors declare that they have no competing interests.

## Authors' contributions

Both XF and JMW were involved in all parts of this work, with XF responsible for experiment design, data collection and data analysis, and JMW advising all these parts. Both authors contributed significantly to the intellectual content of the manuscript and have given final approval of the version to be published.
